# Experimental infection with the hookworm, *Necator americanus*, is associated with stable gut microbial diversity in human volunteers with relapsing multiple sclerosis

**DOI:** 10.1186/s12915-021-01003-6

**Published:** 2021-04-14

**Authors:** Timothy P. Jenkins, David I. Pritchard, Radu Tanasescu, Gary Telford, Marina Papaiakovou, Riccardo Scotti, Alba Cortés, Cris S. Constantinescu, Cinzia Cantacessi

**Affiliations:** 1 Department of Veterinary Medicine, University of Cambridge, Cambridge, UK; 2 Present address: Department of Biotechnology and Biomedicine, Technical University of Denmark, Kongens Lyngby, Denmark; 3 Department of Pharmacy, University of Nottingham, Nottingham, UK; 4 Division of Clinical Neurology, School of Clinical Sciences, University of Nottingham, Queen’s Medical Centre, Nottingham, UK; 5 National History Museum, London, UK; 6 Departament de Farmàcia i Tecnologia Farmacèutica i Parasitologia, Facultat de Farmàcia, Universitat de València, València, Spain

**Keywords:** Relapsing multiple sclerosis, Hookworm, Helminth therapy, Bacterial 16S rRNA sequencing, Bacterial richness, Bacterial diversity, Relapse, *Parabacteroides*

## Abstract

**Background:**

Helminth-associated changes in gut microbiota composition have been hypothesised to contribute to the immune-suppressive properties of parasitic worms. Multiple sclerosis is an immune-mediated autoimmune disease of the central nervous system whose pathophysiology has been linked to imbalances in gut microbial communities.

**Results:**

In the present study, we investigated, for the first time, qualitative and quantitative changes in the faecal bacterial composition of human volunteers with remitting multiple sclerosis (RMS) prior to and following experimental infection with the human hookworm, *Necator americanus* (*N+*), and following anthelmintic treatment, and compared the findings with data obtained from a cohort of RMS patients subjected to placebo treatment (*PBO*). Bacterial 16S rRNA high-throughput sequencing data revealed significantly decreased alpha diversity in the faecal microbiota of *PBO* compared to *N+* subjects over the course of the trial; additionally, we observed significant differences in the abundances of several bacterial taxa with putative immune-modulatory functions between study cohorts. *Parabacteroides* were significantly expanded in the faecal microbiota of *N*+ individuals for which no clinical and/or radiological relapses were recorded at the end of the trial.

**Conclusions:**

Overall, our data lend support to the hypothesis of a contributory role of parasite-associated alterations in gut microbial composition to the immune-modulatory properties of hookworm parasites.

**Supplementary Information:**

The online version contains supplementary material available at 10.1186/s12915-021-01003-6.

## Background

A growing body of evidence supports a key role of infections by gastrointestinal (GI) helminth parasites in shaping the composition and function of the human gut microbiota, with significant implications for local and systemic host immunity, and metabolic potential (reviewed by [1]). Notably, helminth-driven quantitative and qualitative modifications in the overall make-up of gut microbial populations have been proposed to contribute to the immune-suppressive properties of parasites [2–7]. For instance, in a milestone study conducted by Broadhurst et al. [8], experimental infections of a primate model of chronic idiopathic diarrhoea (CID) with the human large intestinal whipworm, *Trichuris trichiura*, were followed by a significant improvement of clinical signs and weight gain; these were accompanied by a notable increase in microbial alpha diversity in the colonic mucosal microbiota of worm-infected macaques. This observation led the authors to speculate that the onset of Th2-mediated host immune responses against the parasites might have resulted in significant changes of the mucosal environment, such as a reduced bacterial attachment to the intestinal mucosa post-worm colonisation and contractions of populations of potentially pathogenic bacteria to levels comparable to those of healthy controls [8].

Another study conducted in a cohort of human volunteers with coeliac disease (CeD) experimentally infected with the hookworm of the small intestine, *Necator americanus*, reported increases in gut bacterial richness (observed in both faecal samples and biopsy tissues) that followed parasite colonisation and subsequent challenge with increasing doses of gluten [4, 5, 9]; whilst the detected differences did not reach statistical significance (likely due to sample size limitations), the increased gluten tolerance observed in infected CeD volunteers was postulated to result from the anti-inflammatory properties of *N. americanus* partly *via* the restoration of microbial and immune homeostasis [5, 9]. These data point towards a possible role of helminth-associated changes in gut microbial community composition and function in parasite-mediated suppression of chronic inflammation; nonetheless, thus far, studies of the role(s) that the gut microbiota plays in the therapeutic properties of helminth parasites have been carried out in human volunteers with chronic inflammatory gut diseases [5, 9]. Hence, the dysbiotic state of the gut microbiota of these individuals at baseline makes the determination of the mechanisms of microbiota-driven helminth immune-suppression challenging.

Nevertheless, recently, the therapeutic properties of controlled infections by *N. americanus* have been investigated in a double-blinded, randomised, placebo-controlled clinical trial conducted in a cohort of 71 human patients with relapsing multiple sclerosis (RMS) (i.e. Worms for Immune Regulation in Multiple Sclerosis, WIRMS; NCT01470521 [10];). MS is an autoimmune disease of the central nervous system (CNS) characterised by inflammation, demyelination, and subsequent neural damage (reviewed by [11]). Current, long-term immune-suppressive therapies for MS are often associated with severe side effects, and patients will often experience substantial and tragic neurological disabilities related to the disease (reviewed by [11]). The ability of *N. americanus* to stimulate a systemic Th2-dominated environment in the human host represented the main rationale of this clinical trial [12]. Notably, the clinical outcome of the WIRMS study provided further support to the promise of helminth-based therapy for treatment of RMS; indeed, at the end of the study, 51% (*n* = 18/35) of RMS patients experimentally infected with *N. americanus* showed no detectable new CNS lesions, as assessed by magnetic resonance imaging (MRI) scans, vs. 28% (*n* = 10/36) of placebo-treated volunteers [10]. In particular, the percentages of eosinophils and of CD4+CD25^high^CD127^neg^T cells in peripheral blood of worm-colonised individuals was significantly increased 9 months post-infection compared to placebo-treated subjects [10].

Given the existence of robust communications between the gut and the CNS by means of immunological, neural, and endocrine mechanisms (i.e. gut-systemic-CNS axis), as well as recent evidence of an association between gut microbiome phenotype and the onset of MS [13], it is conceivable that the beneficial properties of *N. americanus* in RMS might be linked, at least in part, to the direct or indirect effects that the parasites exert on the composition of the gut microbiota and relative abundances of individual bacterial species. Thus, building on the availability of unique biological specimens (i.e. faecal samples) collected over the course of the WIRMS trial, we explore, for the first time, the longitudinal changes in faecal bacterial profiles of human volunteers with RMS, prior to and following experimental infection with *N. americanus*, and subsequent administration of anthelmintic treatment, and compare the findings with data obtained from a cohort of uninfected, placebo-treated RMS patients. In particular, we show that, unlike the faecal bacterial microbiota of placebo-treated RMS patients, that of *N. americanus*-infected volunteers was characterised by unaltered diversity throughout the course of the trial, and by a significant expansion in populations of bacteria with known immune-modulatory properties (e.g. Tenericutes/Mollicutes) with potential roles in parasite-mediated suppression of autoimmunity.

## Results

### Sequencing output and faecal bacterial profiles of parasite-infected *vs*. placebo-treated RMS patients

**Fig. 1:**
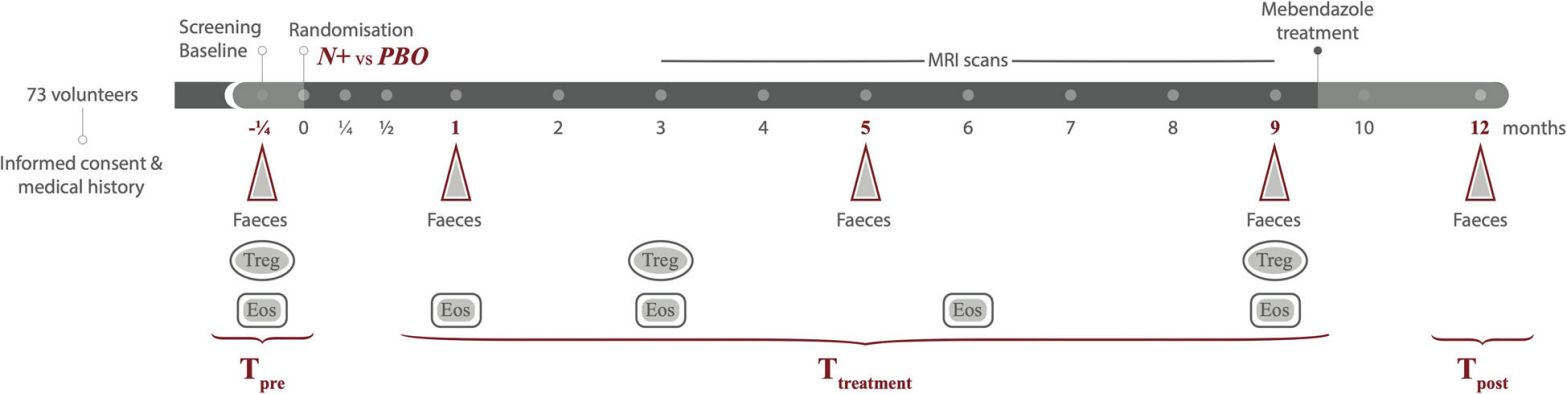
Overview of the clinical study design. A total of 73 volunteers suffering from relapsing multiple sclerosis (RMS) were included in the trial and randomly assigned to the two treatment arms, i.e. percutaneous infection with 25 *N. americanus* larvae (*N*+), or placebo treatment with pharmacopoeial grade water (*PBO*). Triangles indicate the timepoints corresponding to faecal sample collection for metagenomic sequencing, whilst ellipses and rectangles indicate collection of blood samples for assessment of regulatory T cell (Treg) and eosinophil (Eos) counts, respectively (data available from [10]). Samples collected prior to hookworm infection = T_pre_; samples collected at 1, 5, and 9 months post-infection/placebo treatment = T_treatment_; samples collected post-anthelmintic treatment = T_post_

This longitudinal study relied on the availability of faecal samples collected at selected time points throughout the WIRMS trial, i.e. 1 week prior to experimental infection/placebo treatment (p.i./p.; T_pre_), and at 1, 5 and 9 months p.i./p. (T1, T5 and T9, respectively; samples together referred to as ‘T_treatment_’) and 2 months post-anthelmintic treatment (T_post_; Fig. 1). Fifty WIRMS study subjects (i.e. 36 females and 14 males), out of 71 patients who completed the clinical trial, provided faecal samples at these time points and were thus included in this study (see Fig. 1, Table 1, Materials and Methods, and [10]). A total of 250 faecal samples were analysed for bacterial profiling as described below. Following data and metadata unblinding at the end of the trial (cf. [10]), it was established that 24 of these subjects had been percutaneously infected with 25 *N. americanus* infective larvae (*N+*, 17 females and 7 males), whilst 26 had been placebo-treated with pharmacopoeial grade water (= *PBO*) (19 females and 7 males). High-throughput (Illumina) sequencing of the bacterial 16S rRNA gene fragment performed on 226 DNA extracts (out of initial 250) yielded a total of 16,158,693 (per sample mean: 68180 ± 70,000) paired-end reads; of these, 9,100,255 high-quality sequences (per sample mean 38,397 ± 31,519) were retained following quality control. Rarefaction curves generated following in silico subtraction of low-quality sequences indicated that the majority of faecal bacterial communities were represented in the remaining sequence data, thus allowing us to undertake further analyses (data available from [14]).

**Table 1:** Demographic characteristics and clinical features of volunteers with relapsing multiple sclerosis (RMS) experimentally infected with the hookworm, *Necator americanus* (*N*+), or placebo-treated (*PBO*), included in the present study

Patient characteristics	*N*+ (*n* = 24)	*PBO* (*n* = 26)
*Demographic characteristics*		
Age (years, standard deviation)	45.4 (8.8)	45.8 (10.9)
Gender (no. of subjects, %)		
Female	17 (70.9%)	19 (73.1%)
Male	7 (29.1%)	7 (26.9%)
Ethnicity (white, %)	23 (95.8)	24 (92.3)
*Clinical features*		
Mean EDSS score at T_pre_ (range)	3.1 (1.5–5)	3.1 (1.5–5)
Mean EDSS change during the trial	−0.08	+0.17
Previous DMT		
Glatiramer acetate	1	2
IFN-beta	5	2
DMT in the 90 days prior to T_pre_	0	0
Months from last DMT (average)	96.6	63.3
Prior steroid treatment* (*short course 3–5d)	8	7
Steroids in the 60 days prior to baseline	0	0
Steroids in the 90 days prior to baseline	1	1
Months from last steroid treatment (average)	63.7	112.2
*Clinical and/or radiological relapses*		
Clinical relapses (including MRI activity)	3	5
Radiological relapses (MRI activity only)	7	12

*T*_*pre*_, 1 week prior to experimental hookworm infection/placebo treatment; *EDSS*, Expanded Disability Status Scale; *DMT*, disease-modifying therapies; *MRI*, magnetic resonance imaging

**Fig. 2:**
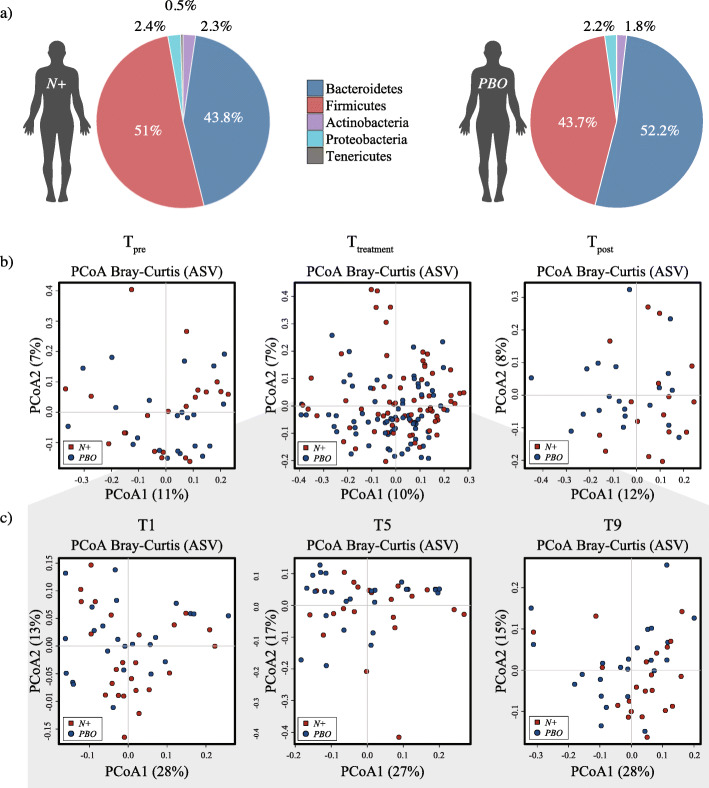
Faecal bacterial profiles of volunteers with relapsing multiple sclerosis (RMS) infected with *Necator americanus* (*N+*) or placebo-treated (*PBO*). **a** Relative abundances of bacterial phyla detected in faecal samples collected over the whole course of the WIRMS trial (from T_pre_ through to T_post_). Percentages in individual pie chart sections indicate the relative proportion of the corresponding phylum. **b** Differences between the faecal bacterial profiles of *N+* and *PBO* subjects 1 week prior to infection/placebo treatment (T_pre_; left), at 1, 5, and 9 months post-infection/placebo treatment (T_treatment_; centre) and 2 months post-anthelmintic treatment (T_post_; right) ordinated by unsupervised principal coordinates analysis (PCoA) at amplicon sequence variant (ASV) level. **c** PCoA analyses of bacterial profiles of samples collected at individual time points within T_treatment_ (i.e. T1, T5 and T9, grey area) from *N*+ and *PBO*

These high-quality sequences were assigned to a total of 5611 amplicon sequence variants (ASVs) and 14 bacterial phyla (data available from [14]). The phyla Bacteroidetes (*N+* = 43.8% average ± 0.4% standard deviation, and *PBO* = 52.2% ± 1.6%, respectively) and Firmicutes (*N+* = 51% ± 0.3%, and *PBO* = 43.7% ± 0.3%, respectively) were predominant in all samples analysed (irrespective of sampling time points), followed by the phyla Proteobacteria (*N+* = 2.4% ± 1.4%, and *PBO* = 2.2% ± 2%, respectively), Actinobacteria (*N+* = 2.3% ± 1.3%, and *PBO* = 1.8% ± 1.2%, respectively), and Tenericutes (*N+* = 0.5% ± 3%, and *PBO* = 0.1% ± 3.1%, respectively; Fig. 2a). Faecal microbial community profiles were ordinated *via* principal coordinates analysis (PCoA) at ASV level; no clear separation between faecal bacterial communities of parasite-infected and placebo-treated RMS volunteers was observed (Fig. 2b and c). Canonical correspondence analysis (CCA) yielded no significant differences between female and male study subjects at T_pre_, individual time points within T_treatment_, and T_post_ (Additional file 1).

### Infection with *Necator americanus* is associated with stable faecal bacterial alpha diversity

**Fig. 3:**
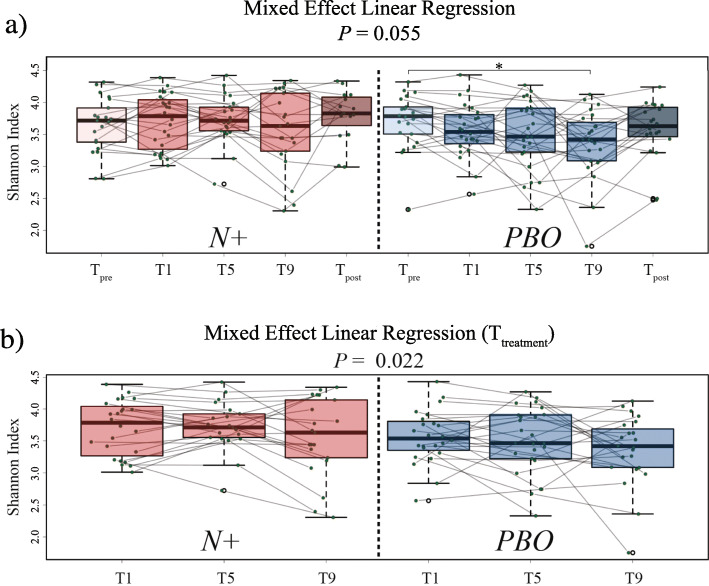
Faecal bacterial alpha diversity of volunteers with relapsing multiple sclerosis (RMS) infected with *Necator americanus* (*N*+) or placebo-treated (*PBO*). **a** Mixed effect linear regression indicating differences in bacterial Shannon diversity within and between *N*+ and *PBO* subjects 1 week prior to infection/placebo treatment (T_pre_), at 1, 5, and 9 months post-infection/placebo treatment (T_treatment_), and 2 months post-anthelmintic treatment (T_post_). **b** Differences in faecal bacterial Shannon diversity between *N*+ (left panel) and *PBO* (right panel) subjects over the course of T_treatment_. *P < 0.05

**Fig. 4:**
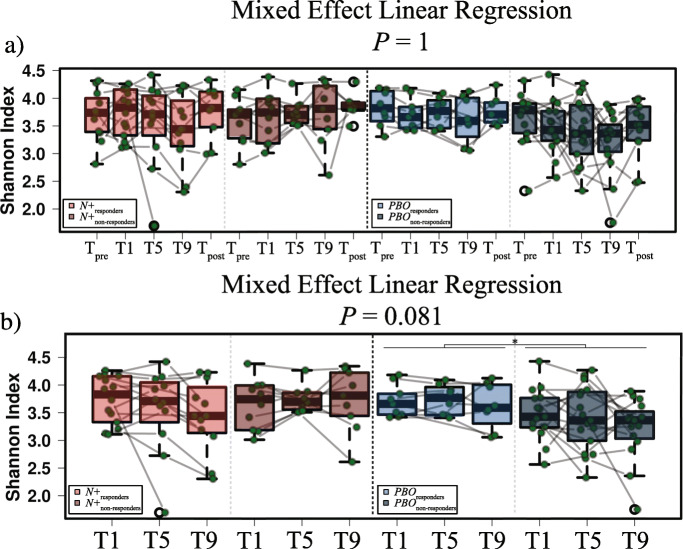
Mixed effect linear regression (MELR) indicating differences in faecal bacterial alpha diversity of volunteers with relapsing multiple sclerosis (RMS) experimentally infected with *Necator americanus* (*N*+), or placebo-treated (*PBO*). **a** Differences in Shannon diversity 1 week prior to infection/placebo treatment (T_pre_), at 1, 5, and 9 months post-infection/placebo treatment (T_treatment_) and 2 months post-anthelmintic treatment (T_post_) are shown for both *N*+ and *PBO* subjects who suffered clinical and/or radiological relapses over the course of the trial (*N*+_non-responders_; *PBO*_non-responders_) *vs*. subjects for which no relapses were recorded (*N*+_responders_; *PBO*_responders_). **b** Differences in faecal bacterial Shannon diversity between *N*+_responders_ and *N*+_non-responders_ (left panel), and *PBO*_responders_ and *PBO*_non-responders_ (right panel) subjects over the course of T_treatment_. *P < 0.05

**Fig. 5:**
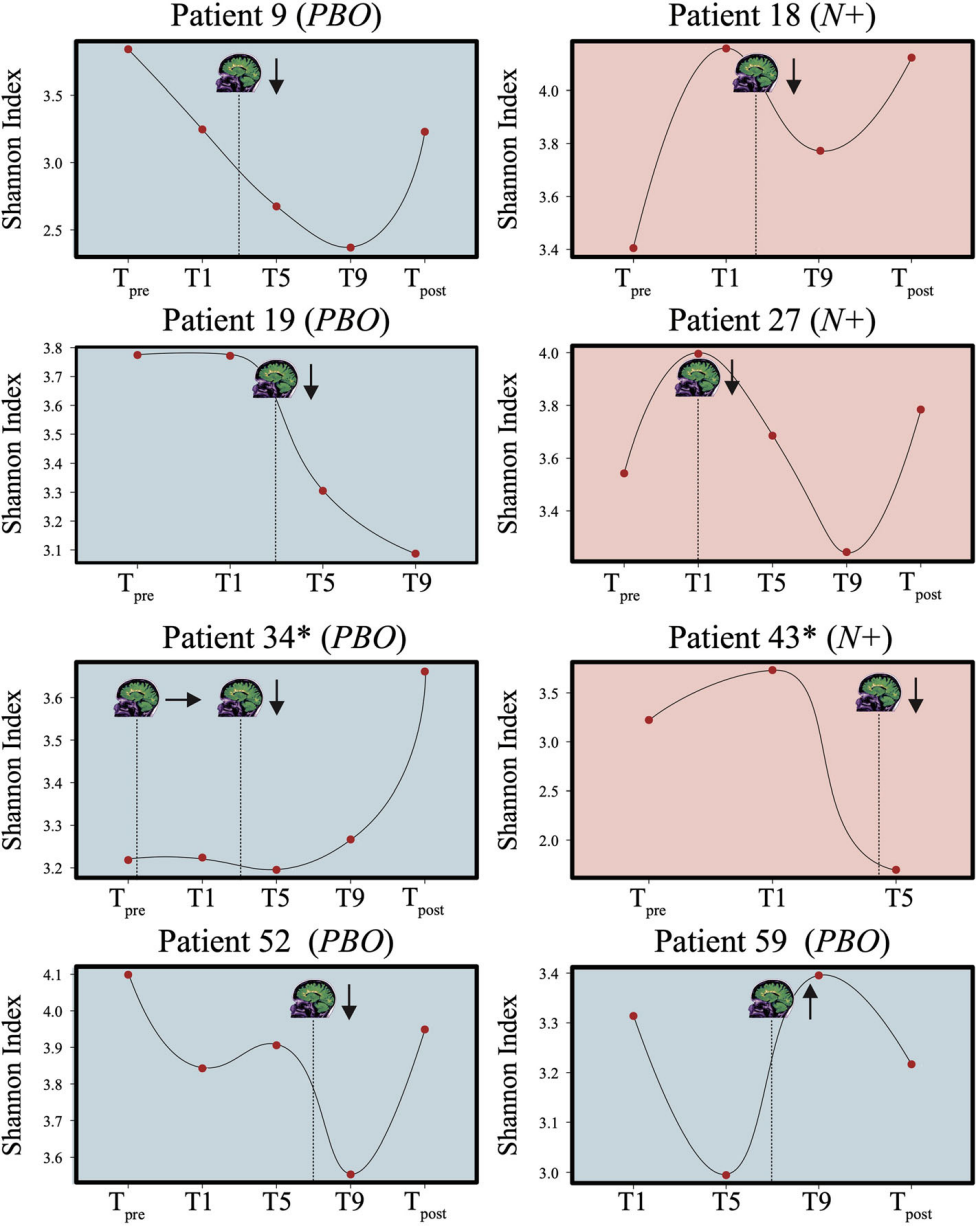
Faecal bacterial alpha diversity of volunteers with relapsing multiple sclerosis (RMS) experimentally infected with *Necator americanus* (*N*+), or placebo-treated (*PBO*). When available, trends in Shannon diversity at 1 week prior to the beginning of the study (T_pre_), at 1, 5, and 9 months post-infection/placebo treatment (T1, T5, and T9, respectively), and 2 months post-anthelmintic treatment (T_post_) are shown for both *N*+ (light red) and *PBO* (blue) subjects who suffered a clinical relapse over the course of the WIRMS trial. The stylised MRI scan indicate the timepoint(s) at which clinical relapses and new MRI lesions were recorded, whilst arrows indicate whether faecal bacterial alpha diversity increased, decreased or remained unaltered following the relapse. Asterisks (*) indicate patients who were administered steroid treatment (methylprednisolone, 2500 to 3000 mg, over 3 to 5 days) following the clinical and radiological relapse

A repeated measures analysis of bacterial alpha diversity was conducted by comparing samples collected at T_pre_ *vs*. 1 month p.i., 1 *vs*. 5 months p.i., 5 *vs*. 9 months p.i., and 9 months p.i. *vs*. T_post_. Overall, no significant differences in bacterial alpha diversity, measured through the Shannon index, were detected between the faecal microbiota of *N+* and *PBO* over the course of the WIRMS trial (*P* = 0.055; Fig. 3a). Nevertheless, alpha diversity was significantly decreased in the bacterial faecal microbiota of *PBO* subjects at T9 compared with T_pre_ (*P* < 0.05; Fig. 3a); this predominantly resulted from decreased microbiota evenness (*P* = 0.036), rather than richness (*P* = 0.09; Additional file 2). However, a significant difference in faecal bacterial alpha diversity was detected between *N*+ and *PBO* subjects over the course of T_treatment_ (*P* = 0.022; Fig. 3b). Additionally, Shannon diversity was significantly decreased in *PBO* patients who had suffered clinical and/or radiological relapses (cf. Materials and methods and [10]) (*PBO*_relapse_ = 5, and *PBO*_MRI-active_ = 12; together referred to as *PBO*_non-responders_) compared to patients who suffered no relapses (*PBO*_responders_ = 9) (*P* < 0.05) (Figs. 4 and 5). No significant differences in Shannon diversity were recorded between *N*+ patients who had suffered relapses (*N+*_relapse_ = 3 and *N+*_MRI-active_ = 7; together referred to as *N+*_non-responders_) compared to patients who suffered no relapses (*N+*_responders_ = 14) (Fig. 4). Significant differences in gut microbial beta diversity between *N+* and *PBO* patients were only detected at T9, with higher beta diversity in *N+* compared to *PBO* subjects (*P* = 0.048; Additional file 3).

### Tenericutes/Mollicutes are expanded in the faecal microbiota of parasite-infected RMS volunteers

**Fig. 6:**
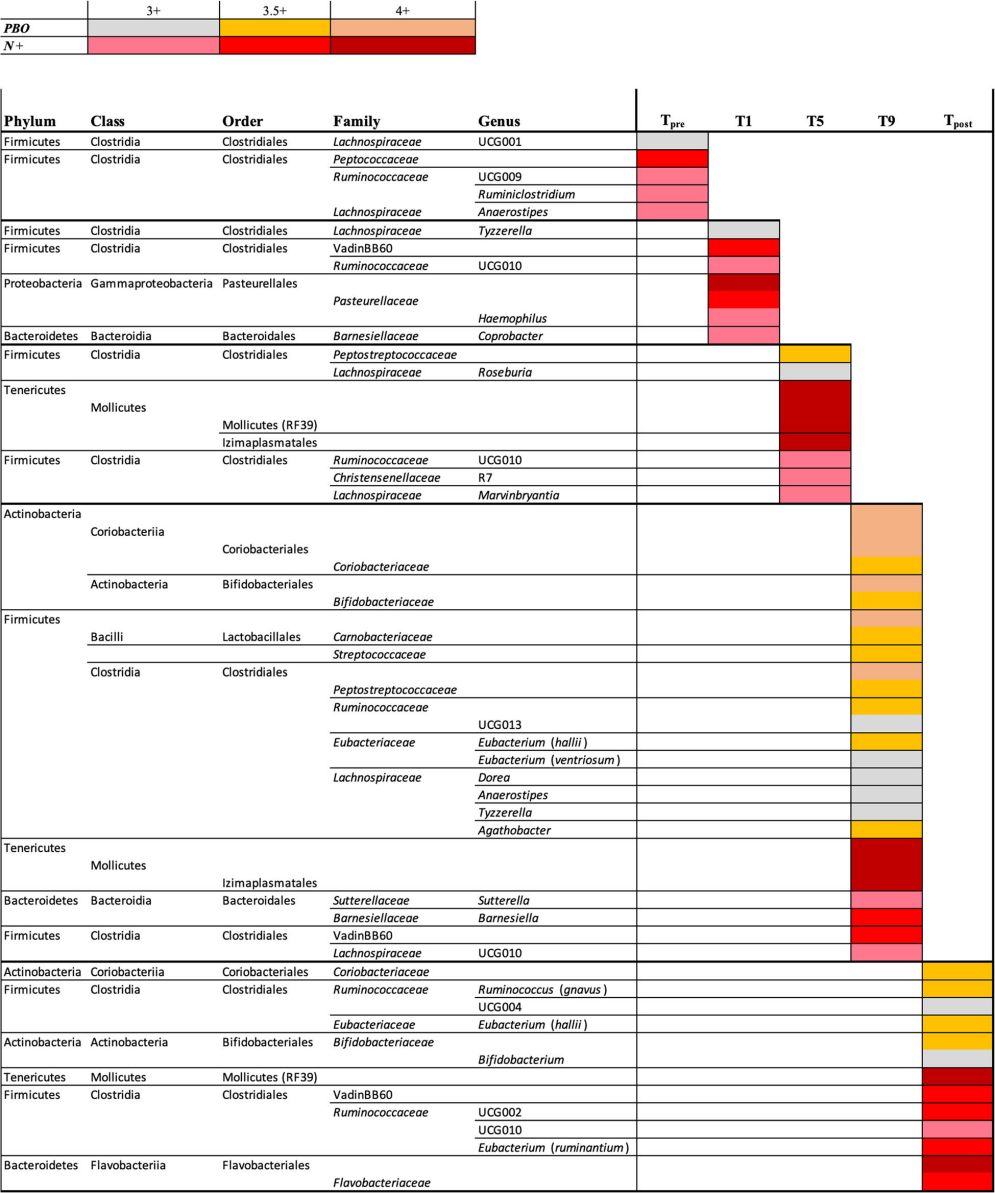
Differentially abundant bacterial taxa in the faecal microbiota of volunteers with relapsing multiple sclerosis (RMS) infected with *Necator americanus* (*N+*) or placebo-treated (*PBO*), 1 week prior to infection/placebo treatment (T_pre_), at 1, 5, and 9 months post-infection/placebo treatment (T_treatment_), and 2 months post-anthelmintic treatment (T_post_) based on linear discriminant analysis effect size (LEfSe) analysis. Colours correspond to linear discriminant analysis (LDA) scores of 4 or higher (*N+* = dark red; *PBO* = ochre), 3.5 to 4 (*N+* = red; *PBO* = orange), and 3 to 3.5 (*N+* = pink; *PBO* = grey)

Linear discriminant analysis effect size (LEfSe) analysis revealed significant differences in the relative abundances of individual bacterial taxa (phylum to ASV level) between *N+* and *PBO* patients at T_pre_, individual time points within T_treatment_, and T_post_ (Fig. 6). Of these bacterial taxa, five genera (*Roseburia*, *Dorea*, *Tyzzerella*, *Anaerostipes*, and *Agathobacter*) belonging to the family *Lachnospiraceae*, *Peptostreptococcaceae*, *Carnobacteriaceae*, and *Coriobacteriaceae* were significantly more abundant in *PBO* compared to *N+* subjects over the course of T_treatment_ (Fig. 6). Conversely, amongst other bacterial groups, two orders (RF39 and Izimaplasmatales) belonging to Tenericutes/Mollicutes were significantly more abundant in the faecal microbiota of *N+* subjects than in that of *PBO* (Fig. 6). Notably, differences in Mollicutes abundance resulted from expanded populations of these bacteria in the microbiota from *N+* and a simultaneous contraction of the same taxa in faecal samples from *PBO* (Additional file 4). Finally, *Coriobacteriaceae* remained more abundant in the microbiota of *PBO* subjects at T_post_, whilst Tenericutes/Mollicutes remained more abundant in *N+* subjects at the same time point (Fig. 6).

A summary of the findings from this study, including populations of faecal bacteria expanded or reduced upon experimental infection with *N. americanus*, as well as fluctuations in faecal bacterial alpha- and beta diversity over the course of the WIRMS trial, is available from the MICrobiome HELminth INteraction database (MICHELINdb) at http://helminthsandmicrobes.vet.cam.ac.uk [15].

### Relapses are associated with differences in the abundances of selected bacterial populations in parasite-infected RMS volunteers

**Fig. 7:**
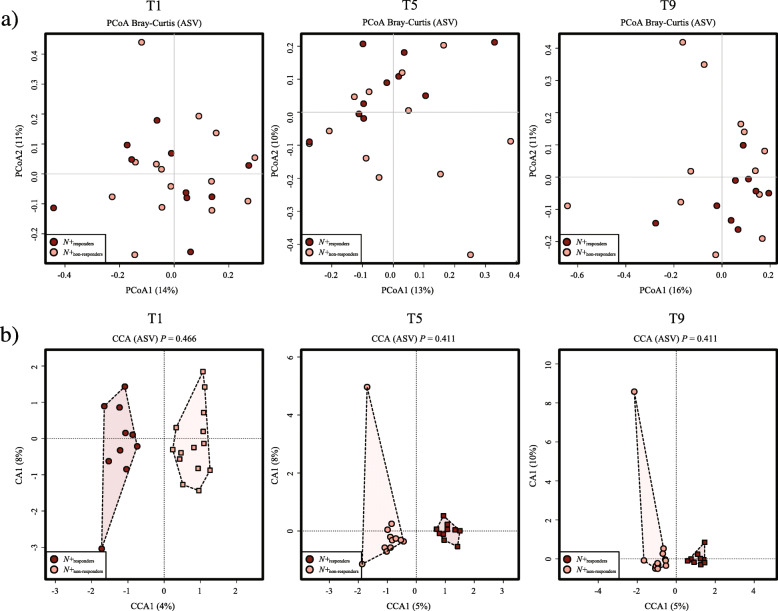
Differences in faecal bacterial profiles between *Necator americanus*-infected volunteers with relapsing multiple sclerosis (RMS) at 1, 5, and 9 months post-infection (T_treatment_), who suffered a clinical and/or radiological relapse during the course of the trial (*N*+_non-responders_) *vs*. volunteers for which no relapses were recorded (*N*+_responders_), investigated *via* **a** principal coordinates analysis (PCoA) and **b** canonical correspondence analysis (CCA)

**Fig. 8:**
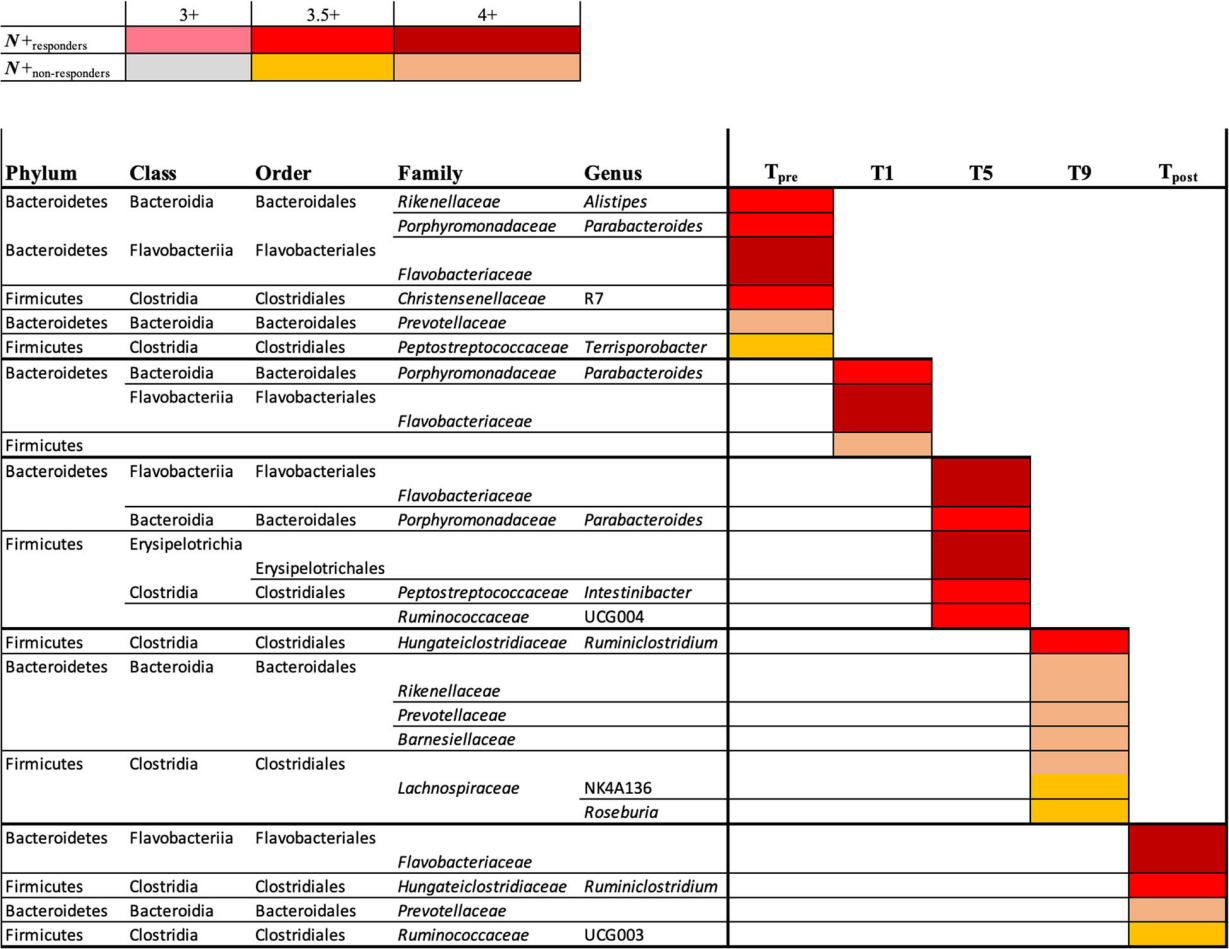
Differentially abundant bacterial taxa between *Necator americanus*-infected volunteers with relapsing multiple sclerosis (RMS) who suffered a clinical and/or radiological relapse (*N*+_non-responders_) *vs*. volunteers for which no relapses were recorded (*N*+_responders_), 1 week prior to infection/placebo treatment (T_pre_), at 1, 5, and 9 months post-infection/placebo treatment (T_treatment_), and 2 months post-anthelmintic treatment (T_post_) based on linear discriminant analysis effect size (LEfSe) analysis. Colours correspond to linear discriminant analysis (LDA) scores of 4 or higher (*N*+_responders_ = dark red; *N*+_non-responders_ = ochre), 3.5 to 4 (*N*+_responders_ = red; *N*+_non-responders_ = orange), and 3 to 3.5 (*N*+_responders_ = pink; *N*+_non-responders_ = grey)

**Fig. 9:**
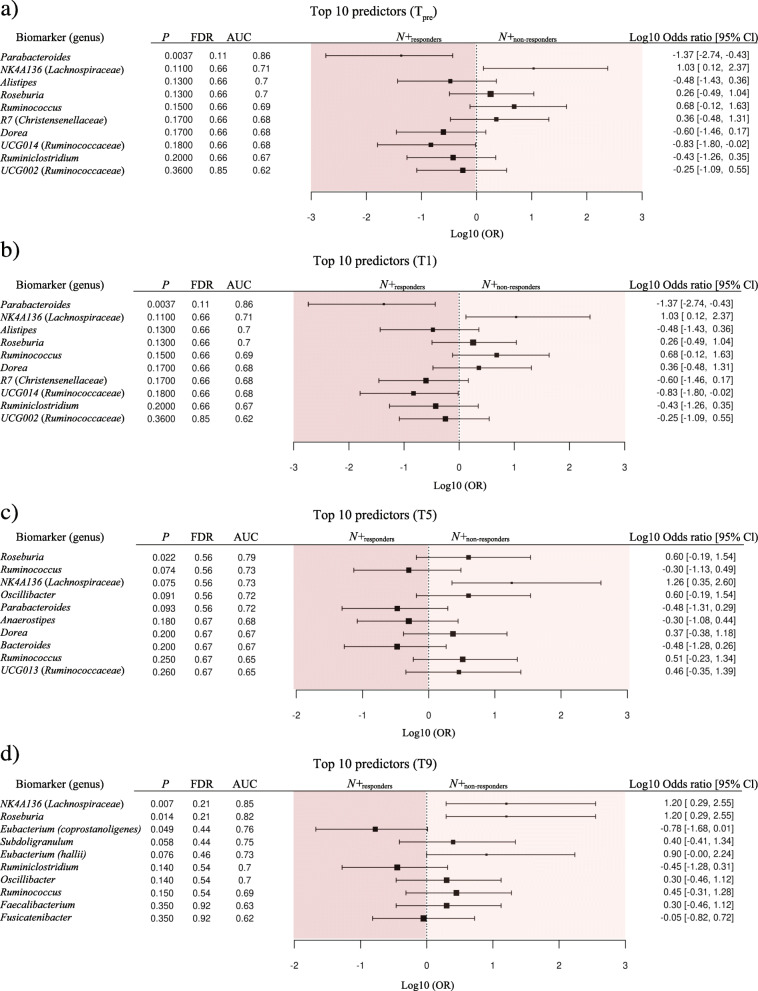
Top 10 faecal bacterial taxa whose relative abundances were identified as putative biomarkers of positive (*N+*_responders_) or negative (*N*+_non-responders_) clinical outcome for *Necator americanus*-infected patients (*N+*) over the course of the WIRMS trial. Biomarker predictions were conducted **a** 1 week prior to infection/placebo treatment (T_pre_) and at **b** 1, **c** 5, and **d** 9 months post-infection (T_treatment_)

A proportionally smaller number of *N*+ and *PBO* individuals who presented clinical relapses and/or significant MRI activity throughout the WIRMS trial [*N*+_non-responders_ = 10 of 17 (59%) and *PBO*_non-responders_ = 17 of 26 (65%)] provided longitudinal faecal samples for microbiota analysis compared with individuals who did not show disease activity [*N*+_responders_ = 14 of 18 (78%) and *PBO*_responders_ = 9 of 10 (90%)] (cf. [10]). Differences in faecal bacterial composition between *N*+_non-responders_ (*n* = 10) *vs*. *N*+_responders_ (*n* = 14) for which samples were available were therefore investigated. No substantial differences in overall bacterial composition were detected between faecal samples from these sub-cohorts at T_pre_ and T_post_ (Additional file 5), as well as at individual time points within T_treatment_ *via* PCoA and CCA (Fig. 7). No significant differences in Shannon and beta diversity were detected between these groups at any timepoint (Fig. 4 and Additional file 6). However, within *PBO*, Shannox index was significantly decreased in *PBO*_non-responders_ compared with *PBO*_responders_ over the course of T_treatment_ (Fig. 4). Differences in the relative abundances of individual bacterial taxa (phylum to species level) between the faecal microbiota of *N*+_non-responders_ and *N*+_responders_ were determined via LEfSe (Fig. 8). Amongst other bacterial taxa, *Porphyromonadaceae* (*Parabacteroides*) were significantly more abundant in the faecal microbiota of *N*+_responders_, compared to that of *N*+_non-responders_ at T1 and T5 (Fig. 8), whilst the opposite trend was observed for taxa within *Rikenellaceae*, *Lachnospiraceae* (*Roseburia* and NK4A136), *Barnesiellaceae* and *Prevotellaceae* at T9 (Fig. 8). Analysis of bacterial taxa associated with a positive outcome of hookworm treatment in RMS, yielded levels of *Parabacteroides* as the best predictor for *N*+_responders_ (high levels) and *N*+_non-responders_ (low levels) cohorts at T_pre_ (Fig. 9). *Parabacteroides* also remained the best predictor at T1, whilst the abundances of *Roseburia* and *Eubacterium* (*coprostanoligenes*) were associated with negative hookworm treatment outcome in RMS patients at T5 and T9, respectively (Fig. 9). Conversely, high levels of Ruminococcaceae (UCG005) were associated to the occurrence of clinical and/or radiological relapses in *PBO* at T_pre_ (Additional file 7).

## Discussion

In the present MHRA-approved study, we investigated, for the first time, the quantitative and qualitative changes in faecal bacterial profiles of human volunteers with RMS prior to and following experimental infection with *N. americanus*, and following administration of anthelmintic treatment, and compared the findings with data obtained from an age- and gender-matched cohort of RMS patients subjected to placebo treatment.

### Changes in faecal bacterial diversity

Bacterial alpha diversity was significantly higher in faecal samples from *N+* subjects compared to *PBO* over the course of helminth treatment. It must however be pointed out that this observation was predominantly linked to a marked decrease of alpha diversity, and specifically of bacterial evenness, in *PBO* over time. This finding is of particular interest, since elevated levels of microbial alpha diversity are typically associated with a ‘healthier’ gut microbiome and overall host health (reviewed by [16]). Similarly, increases in gut microbial alpha diversity were reported in CeD subjects experimentally infected with *N. americanus*, which led the authors to speculate that this mechanism might be (at least partially) responsible for the therapeutic effect of deliberate helminth infections in individuals affected by selected allergic and autoimmune disorders [5, 9]. On the other hand, a decrease in gut microbial alpha diversity has previously been reported during MS relapses [17]. This matches our observations of a significantly lower bacterial alpha diversity being detected in the faecal microbiota of *PBO* volunteers who suffered clinical and/or radiological relapses over the course of T_treatment_ (cf. Fig. 4) as well as decreased alpha diversity following specific clinical relapse events in both *N+* and *PBO* patients (cf. Fig. 5). Within the latter subgroups, two patients (i.e. Patient 43 and 34 in the *N*+ and *PBO* group, respectively), were administered methylprednisolone following clinical relapses. Notably, faecal alpha diversity increased following steroid administration in patient 34, whilst samples collected at T9 and T_post_ from patient 43 did not yield usable sequence data (not shown). However, the small number of patients in these subgroups prevents us from further speculating on the causal links between gut bacterial alpha diversity, steroid administration, and disease progression.

### Bacterial taxa expanded in the *PBO* cohort

The relative abundances of several faecal bacterial taxa were also significantly altered in *N*+ subjects compared with the *PBO* cohort, both prior to *N. americanus* experimental infection and, more markedly, post-helminth colonisation. Indeed, despite subject randomisation prior to the beginning of the study, we detected minor differences in faecal bacterial composition between study cohorts at T_pre_. No characterisation of faecal bacterial profiles was conducted prior to group assignments; however, due to the intrinsic heterogeneity of gut microbial communities across any given population [18, 19], differences in the abundances of gut microbial taxa are frequently detected between groups of individuals enrolled in randomised, double-blinded, placebo-controlled trials [20, 21], with varying functional significance [21]. Nevertheless, in our study, most differences between *N+* and *PBO* faecal bacterial profiles were detected post-infection, peaking at 9 months post-helminth colonisation (cf. Fig. 6). Notably, in *PBO* patients, bacterial taxa that have been previously associated with the gut microbiota of relapsing MS patients [17] were significantly expanded; in particular, a family of highly abundant anaerobic bacteria, the *Lachnospiraceae*, including the genera *Roseburia*, *Dorea*, and *Tyzzerella* (amongst others), were significantly increased in the faecal microbiota of *PBO* compared to *N+* subjects post-infection. *Lachnospiraceae* is a key family of the human gut microbiome that degrades complex polysaccharides to short-chain fatty acids (SCFAs). These SCFAs are known for their anti-inflammatory properties [22]. This is of note, since *Lachnospiraceae* have been reported to be substantially expanded in the gut microbiota of individuals affected by pathological conditions, such as inflammatory bowel disease (IBD) [23]. The expansion of *Lachnospiraceae* and its respective genera in the *PBO* cohort over the course of the trial is likely associated to MS disease progression and an immune shift towards a pro-inflammatory phenotype [24]. Of note, the abundance of *Lachnospiraceae* was negatively associated with human infections with soil-transmitted helminths (including *Necator*) [25]. However, in hamsters infected with *Ancylostoma ceylanicum*, the abundance of *Lachnospiraceae* in parasite-colonised *vs*. uninfected animals was inconsistent, with *Coprococcus* and *Clostridium XIVb*, and *Roseburia* and *Acetatifactor*, positively and negatively associated with worm infection, respectively [26].

### Bacterial taxa expanded in the *N+* cohort

Bacteria belonging to the low abundant phylum Tenericutes were substantially and consistently increased in the faecal microbiota of *N+* patients post-infection. This phylum consists of the sole class Mollicutes, Gram-negative, small and wall-less bacteria that fulfil a diverse array of roles within the mammalian microbiome (reviewed by [27, 28]). The relative abundance of these bacteria has been reported to differ between the normal gut microbiota and that featuring in a wide range of autoimmune conditions, such as IBD [29, 30], type 1 diabetes [31–33], MS [34], and experimental autoimmune encephalomyelitis (EAE; a murine model of MS) [35]. Albeit inconsistently [32, 33], Tenericutes/Mollicutes are often reduced in the gut microbiota of the diseased cohort, when compared to healthy controls [29–31, 34, 35]. Notably, whilst one of these studies reported higher Tenericutes abundance in the gut microbiota of healthy controls than in paediatric MS patients, exposure to immune-modulatory drugs reversed this trend [34]. Indeed, these bacteria have been suggested to proliferate in Th2-dominant environments [8, 36]. Tenericutes/Mollicutes were also expanded in the gut microbiota of vertebrates infected by Th2-inducing helminth parasites, human cohorts naturally infected with roundworms (i.e. *Trichuris* and/or *Ascaris* and/or hookworm) [37], rats infected with the tapeworm *Hymenolepis diminuta* [36], and primates with CID experimentally infected with *T. trichiura* [8]. This is in accordance with data from the WIRMS trial that reported a markedly increased eosinophilia in *N+* compared to *PBO* subjects [10]. Nevertheless, the functional consequences that expanded populations of Tenericutes/Mollicutes associated to infection by parasitic helminths play in the pathophysiology of the abovementioned chronic inflammatory and autoimmune disorders, as well as in RMS, remains to be determined.

### Faecal bacterial differences between *N+*_responders_ and *N+*_non-responders_

In addition, we investigated the differences in faecal bacterial composition between *N+*_responders_ and *N+*_non-responders_, with the aim to identify potential candidates with roles in MS disease activity. Whilst the overall faecal bacterial composition of these sub-cohorts did not differ substantially prior to helminth infection and post-anthelmintic treatment, differences were recorded post-helminth colonisation. In particular, *Flavobacteriaceae*, a low abundant family in the phylum Bacteroidetes [38], was consistently increased in the faecal microbiota of *N*+_responders_. These bacteria have been repeatedly reported to be depleted in people suffering from autoimmune conditions, such as rheumatoid arthritis [39] and myasthenia gravis [40], although the functional importance of this taxon in the pathophysiology of these conditions remains elusive. Finally, in this study, we asked the question of whether some of the minor differences in faecal bacterial profiles observed between *N+*_responders_ and *N+*_non-responders_ prior to hookworm experimental infection might be associated with positive or negative clinical outcomes. Amongst others, the prevalent genus *Parabacteroides* was significantly more abundant in *N*+_responders_ compared to *N+*_non-responders_ at T_pre_ and identified as the top ranking biomarker of treatment outcome *via* machine learning. Notably, *Parabacteroides* was more abundant in the gut microbiota of *A. ceylanicum*-infected hamsters compared with uninfected counterparts [26]. Furthermore, a recent study conducted in murine models of RMS and chronic-progressive MS identified *Parabacteroides* as more abundant in the gut microbiota of control mice [41]. Additionally, a previous study detected a significant reduction of *Parabacteroides* populations in the faecal microbiota of 71 MS patients not undergoing immune-suppressive treatment compared to that of 71 healthy control subjects [42]. Subsequent monocolonisation of antibiotic-treated mice with *Parabacteroides distasonis* led to significant increases in the CD4+IL-10+ T lymphocyte population in mesenteric lymph nodes and spleens [42]. Furthermore, stimulation of peripheral blood mononuclear cells (PBMCs) from MS patients or healthy controls with total bacterial extracts isolated from the stool samples of the same subjects resulted in the inability of PBMCs from MS patients to differentiate or expand CD25+FoxP3+ Treg populations [42]. This observation led the authors to hypothesise that prior exposure to *P. distasonis* or other ‘beneficial’ bacteria may have contributed to the expanding regulatory T lymphocyte precursor populations in mice, hence promoting anti-inflammatory responses upon subsequent exposure to the same bacteria [42]. However, it is of note that the abundance of *Parabacteroides* in the faecal microbiota of *PBO* patients at T_pre_ was not linked to MRI activity, although the method of microbiota profiling used in this study prevents us from providing species-level annotation. Furthermore, interestingly, in the original study by Tanasescu et al. [10], CD4+CD25^high^CD127^neg^ T cells counts (surrogates of suppressor Treg populations) performed on peripheral blood of *N+*_responders_ and *N+*_non-responders_ were not associated to MRI activity.

## Conclusions

Overall, our data lend support to the hypothesis of a contributory role of parasite-associated modulation of host bacterial microbiota composition to the immune-suppressive properties of hookworms and will be of value in future mechanistic studies aimed to investigate the causality of these interactions. Indeed, whether fluctuations of the abundances of individual gut microbial populations are directly linked to parasite establishment (e.g. *via* the activity of worm-secreted antimicrobials) and/or, indirectly, to changes in the immune environment in response to hookworm colonisation (cf. [43]) remains to be established. Such follow-up experiments are likely to be conducted in rodent models of MS experimentally infected with hookworm parasites (i.e. *A. ceylanicum* and/or *Nippostrongylus braziliensis*), which will require careful consideration of initial parasite infection dose that must mimic that used in human volunteers in order to allow meaningful comparisons between findings.

## Materials and methods

### Ethics statement

This phase 2, single centre, randomised, double-blinded, placebo-controlled clinical trial (WIRMS; Clinicaltrials.gov identifier NCT01470521) aimed to assess the therapeutic efficacy of live hookworm (*N. americanus*) infective larvae in patients with RMS [10]. The trial was conducted at the Queen’s Medical Centre, University of Nottingham, UK. The study was approved and carried out in strict accordance and compliance with the National Research Ethics Service Committee East Midlands (reference 11/EM/0140). Written informed consent was obtained from all subjects enrolled in the study.

### Trial design

For details of patient recruitment, inclusion and exclusion criteria, and trial design, we refer to the original publication by Tanasescu et al. [10]. Briefly, a total of 73 clinically stable RMS patients aged 18–64 (51 females and 22 males), who suffered at least one relapse over the prior 12 months or two over the prior 24 months and who were not subjected to immune-modulatory treatment were randomised and assigned to the two treatment groups, i.e. percutaneous infection with 25 *N. americanus* infective third-stage larvae (*N+*; *n* = 36), or placebo treatment with pharmacopoeial grade water (*PBO*; *n* = 37) (Fig. 1). Stool samples were collected 1 week prior to infection/placebo-treatment (= T_pre_), as well as 1 (T1), 5 (T5), and 9 (T9) months post-infection/placebo-treatment (together referred to as T_treatment_), and 2 months post-anthelminthic treatment (= T_post_) (Fig. 1). In particular, all participants were provided with kidney basins, sterile gloves and disposable wooden spatulas, and sterile 120-ml collection tubes. Once collected, samples were transported to the laboratory within 4 h, aliquoted into cryotubes and stored at − 80 °C until DNA extraction. Only study subjects who provided samples for all of these time points were included in this study (*n* = 50). Infections were confirmed for each *N*+ patient *via* PCR and qPCR-guided *N. americanus* DNA detection performed using the latest available faecal sample prior to anthelmintic treatment, and following previously established protocols [10, 44, 45]. Patients enrolled in the WIRMS trial were clinically assessed monthly by a neurologist and subjected to MRI in order to record the occurrence of clinical relapses and/or new MRI activity. All clinical relapses were neurologist-confirmed and defined as objective changes on neurological examination resulting in an increased functional status score of the Expanded Disability Status Scale (EDSS) by 2 points, or an increase of the EDSS by one step for EDSS < 5, and 0.5 step for EDSS > 5, respectively [10]. For the purpose of this study, patients who displayed clinical relapses and new MRI activity were defined as ‘*PBO*_relapse_’ and ‘*N+*_relapse_’, for *PBO* and *N*+ respectively, whilst patients who displayed new MRI activity in absence of clinical relapses were defined as ‘*PBO*_MRI-active_’ and ‘*N+*_MRI-active_’_._ Together, *PBO*_relapse_ and *PBO*_MRI-active_, and *N+*_relapse_ and *N+*_MRI-active_, are defined as *PBO*_non-responders_ and *N+*_non-responders_, respectively. ‘*PBO*_responders_’ and ‘*N+*_responders_’ refer to patients in each *PBO* and *N*+ for which no clinical relapse and new MRI activity were recorded over the course of the WIRMS trial. Two volunteers withdrew from the WIRMS trial and thus were excluded from this study.

### DNA extraction and bacterial 16S rRNA Illumina sequencing

Genomic DNA was extracted directly from each faecal sample, using the PowerSoil® DNA Isolation Kit (MO BIO Laboratories, Carlsbad, CA, USA), according to manufacturers’ instructions, within 1 month from sample collection. High-throughput sequencing of the V3-V4 hypervariable region of the bacterial 16S rRNA gene was performed on an Illumina MiSeq platform according to the standard protocols with minor adjustments. Briefly, the V3-V4 region was PCR-amplified using universal primers [46], which contained the Illumina adapter overhang nucleotide sequences, using the NEBNext hot start high-fidelity DNA polymerase (New England Biolabs), 2 ng/μl of template DNA, and the following thermocycling protocol: 2 min at 98 °C, 20 cycles of 15 s at 98 °C – 30 s at 63 °C – 30 s at 72 °C, and a final elongation step of 5 min at 72 °C. Amplicons were purified using AMPure XP beads (Beckman Coulter) and the NEBNext hot start high-fidelity DNA polymerase was used for the index PCR with Nextera XT index primers (Illumina) according to the following thermocycling protocol: 3 min at 95 °C, 8 cycles of 30 s at 95 °C – 30 s at 55 °C – 30 s at 72 °C, and 5 min at 72 °C. The indexed samples were purified using AMPure XP beads, quantified using the Qubit dsDNA high sensitivity kit (Life Technologies), and equal quantities from each sample were pooled. The resulting pooled library was quantified using the NEBNext library quantification kit (New England Biolabs) and sequenced using the v3 chemistry (301 bp paired-end reads). Raw sequence data are available from [14].

### Bioinformatics and statistical analyses

Raw paired-end Illumina reads were trimmed for 16S rRNA gene primer sequences using Cutadapt (https://cutadapt.readthedocs.org/en/stable/) and sequence data were processed using the Quantitative Insights Into Microbial Ecology 2 (QIIME2-2019.1; https://qiime2.org) software suite [47]. Successfully joined sequences were quality filtered, dereplicated, chimeras identified, and paired-end reads merged in QIIME2 using DADA2 [48]. Sequences were clustered into amplicon sequence variants (ASVs) on the basis of similarity to known bacterial sequences available in the SILVA reference database (https://www.arb-silva.de/download/archive/qiime; Silva_132); sequences that could not be matched to references in the SILVA database were clustered de novo based on pair-wise sequence identity (99% sequence similarity cut-off). The first selected cluster seed was considered as the representative sequence of each ASV. The ASV table with the assigned taxonomy was exported from QIIME2 alongside a weighted UniFrac distance matrix. Singleton ASVs were removed prior to downstream analyses. Cumulative-sum scaling (CSS) was applied, followed by log2 transformation to account for the non-normal distribution of taxonomic counts data. Statistical analyses were executed using the Calypso software [49] (cgenome.net/calypso/); samples were ordinated in explanatory matrices using unsupervised PCoA and/or supervised CCA including ‘infection status’ as explanatory variable. Differences in bacterial alpha diversity (Shannon index) between study groups (*N+* and *PBO*) over time were evaluated based on rarefied data (read depth of 8712) and using mixed effect linear regression (MELR). Differences in beta diversity (Bray-Curtis dissimilarity) between *N+* and *PBO* at each time point were identified using analysis of similarity (ANOSIM) and effect size indicated by an *R*-value (between − 1 and + l, with a value of 0 representing the null hypothesis [50]). ANOSIM provides a single *P*-value calculated by comparing intra-group distances (with between-group distances = ‘between’). Pairwise comparisons of microbial communities in samples collected at different time points were carried out using permutational multivariate analysis of variance (PERMANOVA, Bray-Curtis distance) [51], using an additional plugin in QIIME2, i.e. the q2-diversity-plugin, which utilises the beta-group-significance function and correction for multiple testing. Differences in the abundances of individual bacterial taxa between *N+* and *PBO* over time were assessed using the linear discriminant analysis effect size (LEfSe) workflow (LDA effect size score < 2 = discarded; between 3 and 4 = high; > 4 = very high) [52]. LEfSe was also applied to identify differentially abundant groups of bacteria between study subjects associated to positive outcome of hookworm treatment (*N*+_responders_) or displaying MS disease activity (*N*+_non-responders_) over time. In addition, bacterial taxa predictive of treatment outcome were identified *via* Wilcoxon rank test in Calypso [49]. Individual bacteria taxa associated to one of two sample groups (e.g. *N*+_responders_ and *N*+_non-responders_) were detected *via* a support vector machine evaluated by leave-one-out cross-validation [49]. The predictive power of each taxon to discriminate between two sample groups was assessed *via* Wilcoxon rank test (*p* < 0.05), *a*rea *u*nder the receiver operating characteristic (ROC) *c*urve (AUC; 1 = good measure of separability, 0 = poor measure of separability), and odds ratio ([±] 1 = odds of one event being the same in either the presence or absence of the other event, > [±] 1 = the presence of one event increases/reduces the odds of the other event) [49]. This approach embeds the random forest feature selection method and is based on the assumption that the vast majority of faecal bacterial taxa are either redundant (highly correlated) or irrelevant, and can thus be removed without significant loss of information. Random forest identifies the subset of most relevant features (i.e. taxa) by constructing a collection of decision trees. Variance is controlled by constructing trees incorporating only a random subset of these features, which in turn reduces overfitting. The results of the random forest analysis are presented as bar chart, where bars represent putative biomarkers, as estimated by random permutation.

## Supplementary Information

**Additional file 1.** Differences between the faecal bacterial profiles of male and female volunteers with relapsing multiple sclerosis (RMS) at 1 week prior to experimental hookworm infection/placebo treatment (T_pre_), at 1, 5, and 9 months post-infection/placebo treatment (T_treatment_), and 2 months post-anthelmintic treatment (T_post_) ordinated by supervised canonical correspondence analysis (CCA).

**Additional file 2. **Mixed effect linear regression (MELR) indicating differences in faecal bacterial alpha diversity of volunteers with relapsing multiple sclerosis (RMS) experimentally infected with the hookworm, *Necator americanus* (*N*+), or placebo-treated (*PBO*). (a) Differences between faecal bacterial evenness and (b) richness of *N*+ and *PBO* subjects at 1, 5, and 9 months post-infection/placebo treatment (T_treatment_); (c) Differences in faecal microbial evenness and (d) richness between *PBO* subjects prior to and following infection/placebo treatment (T_pre_ and T_treatment_, respectively)

**Additional file 3. **(a) Faecal bacterial beta diversity of volunteers with relapsing multiple sclerosis (RMS) experimentally infected with the hookworm, *Necator americanus* (*N*+), or placebo-treated (*PBO*) 1 week prior to infection/placebo treatment (T_pre_), at 1, 5, and 9 months post-infection/placebo treatment (T1, T5 and T9, respectively), and 2 months post-anthelmintic treatment (T_post_; right). (b) Differences in faecal bacterial beta diversity between time points within *N*+ (left) and *PBO* (right) volunteers over the course of the study. ‘Between’ indicates the difference between groups (i.e. *N*+ and *PBO* [a] and T_pre_, T1, T5, T9 and T_post_ within each *N*+ and *PBO* [b]).

**Additional file 4. **Differences in relative abundance of the bacterial class Mollicutes between the faecal microbiota of volunteers with relapsing multiple sclerosis (RMS) experimentally infected with the hookworm, *Necator americanus* (*N*+), or placebo-treated (*PBO*) across time points determined by ANOVA. Significant differences are indicated by horizontal bars (*p* < 0.05).

**Additional file 5. **Differences in faecal bacterial profiles between *Necator americanus-*infected volunteers with relapsing-multiple sclerosis (RMS) who suffered a clinical and/or radiological relapse (*N*+_non-responders_) *vs*. volunteers for which no relapses were recorded (*N*+_responders_), investigated *via* supervised canonical correspondence analysis (CCA), prior to infection/placebo treatment (T_pre_), as well as post-anthelmintic treatment (T_post_).

**Additional file 6. **Analysis of similarities (ANOSIM) indicating differences in faecal bacterial beta diversity between *Necator americanus-*infected volunteers with relapsing multiple sclerosis (RMS) who suffered a clinical and/or radiological relapse (*N*+_non-responders_) *vs*. volunteers for which no relapses were recorded (*N*+_responders_), 1 week prior to infection/placebo treatment (T_pre_), at 1, 5, and 9 months post-infection (T1, T5 and T9, respectively) and 2 months post-anthelmintic treatment (T_post_). Error bars are also provided. ‘Between’ indicates the difference between groups (i.e. *N*+_responders_ and *N*+_non-responders_).

**Additional file 7. **Top 10 faecal bacterial taxa identified as putative biomarkers of positive (*PBO*_responders_) or negative (*PBO*_non-responders_) clinical outcome for placebo-treated patients (*PBO*) over the course of the WIRMS trial. Biomarker predictions were conducted (a) one week prior to infection/placebo treatment (T_pre_) and at (b) 1, (c) 5, and (d) 9 months post-infection (T_treatment_).

## Data Availability

The bacterial 16S rRNA gene sequence datasets generated and analysed during the current study are available from Mendeley Data (DOI: 10.17632/pkk4vtc57r.1) [14].

## Abbreviations

ANOSIM: Analysis of similarity
CCA: Canonical correspondence analysis
CSS: Cumulative-sum scaling
GI: Gastrointestinal
LDA: Linear discriminant analysis
LEfSe: Linear discriminant analysis effect size
MELR: Mixed effect linear regression
RMS: Relapsing multiple sclerosis
*N*+: Hookworm-infected
ASV: Amplicon sequence variant
PCoA: Principal coordinates analysis
*PBO*: Placebo
