# Supplementary figures and images for: Experimental infection with the hookworm, Necator americanus, is associated with stable gut microbial diversity in human volunteers with relapsing multiple sclerosis
Source: BMC Biol. 2021 Apr 14;19:74. doi: 10.1186/s12915-021-01003-6 (PMC8048248; doi:10.1186/s12915-021-01003-6)

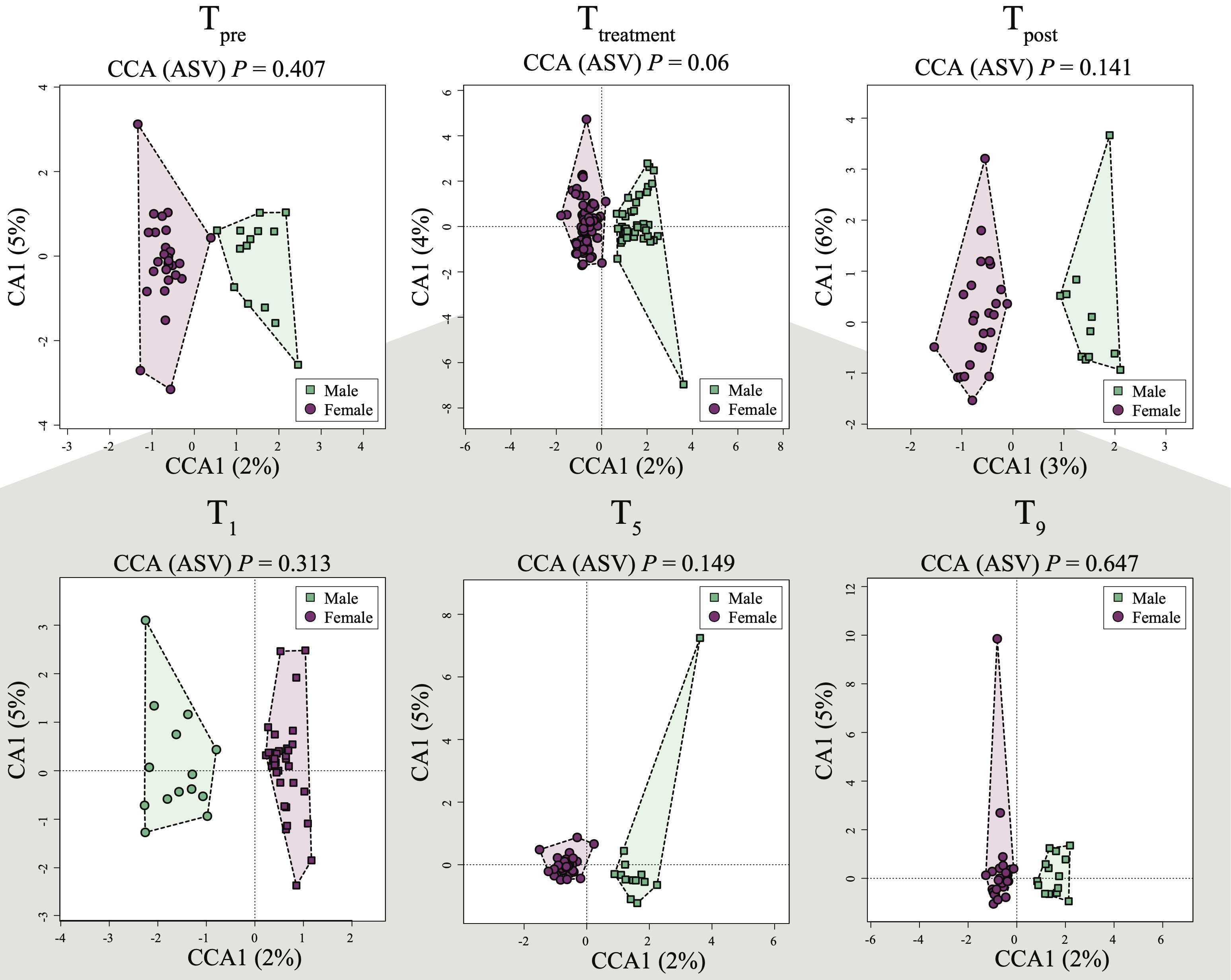

Supplement: Supplementary file 1 — Additional file 1. Differences between the faecal bacterial profiles of male and female volunteers with relapsing multiple sclerosis (RMS) at 1 week prior to experimental hookworm infection/placebo treatment (Tpre), at 1, 5, and 9 months post-infection/placebo treatment (Ttreatment), and 2 months post-anthelmintic treatment (Tpost) ordinated by supervised canonical correspondence analysis (CCA). [file 12915_2021_1003_MOESM1_ESM.jpg]

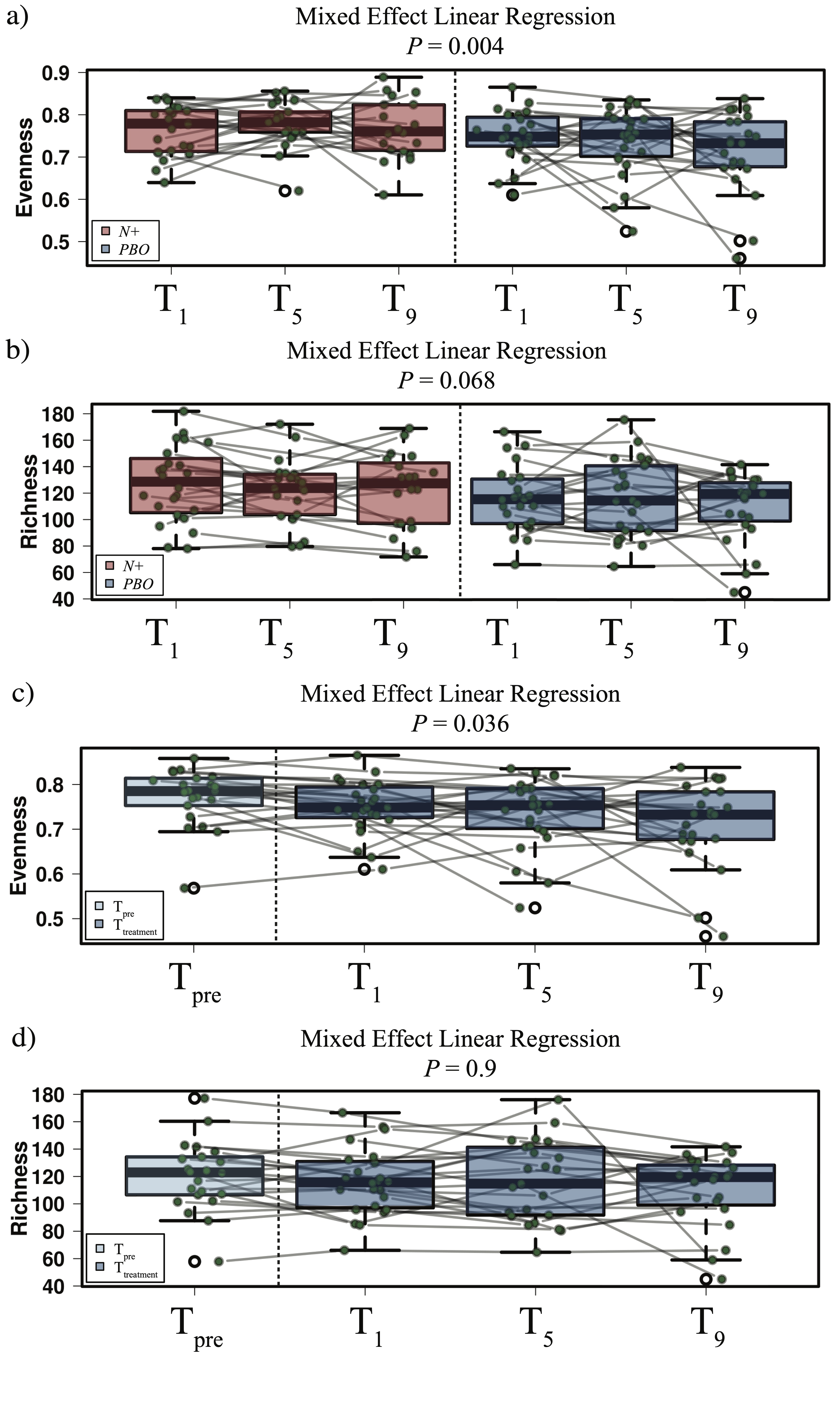

Supplement: Supplementary file 2 — Additional file 2. Mixed effect linear regression (MELR) indicating differences in faecal bacterial alpha diversity of volunteers with relapsing multiple sclerosis (RMS) experimentally infected with the hookworm, Necator americanus (N+), or placebo-treated (PBO). (a) Differences between faecal bacterial evenness and (b) richness of N+ and PBO subjects at 1, 5, and 9 months post-infection/placebo treatment (Ttreatment); (c) Differences in faecal microbial evenness and (d) richness between PBO subjects prior to and following infection/placebo treatment (Tpre and Ttreatment, respectively) [file 12915_2021_1003_MOESM2_ESM.jpg]

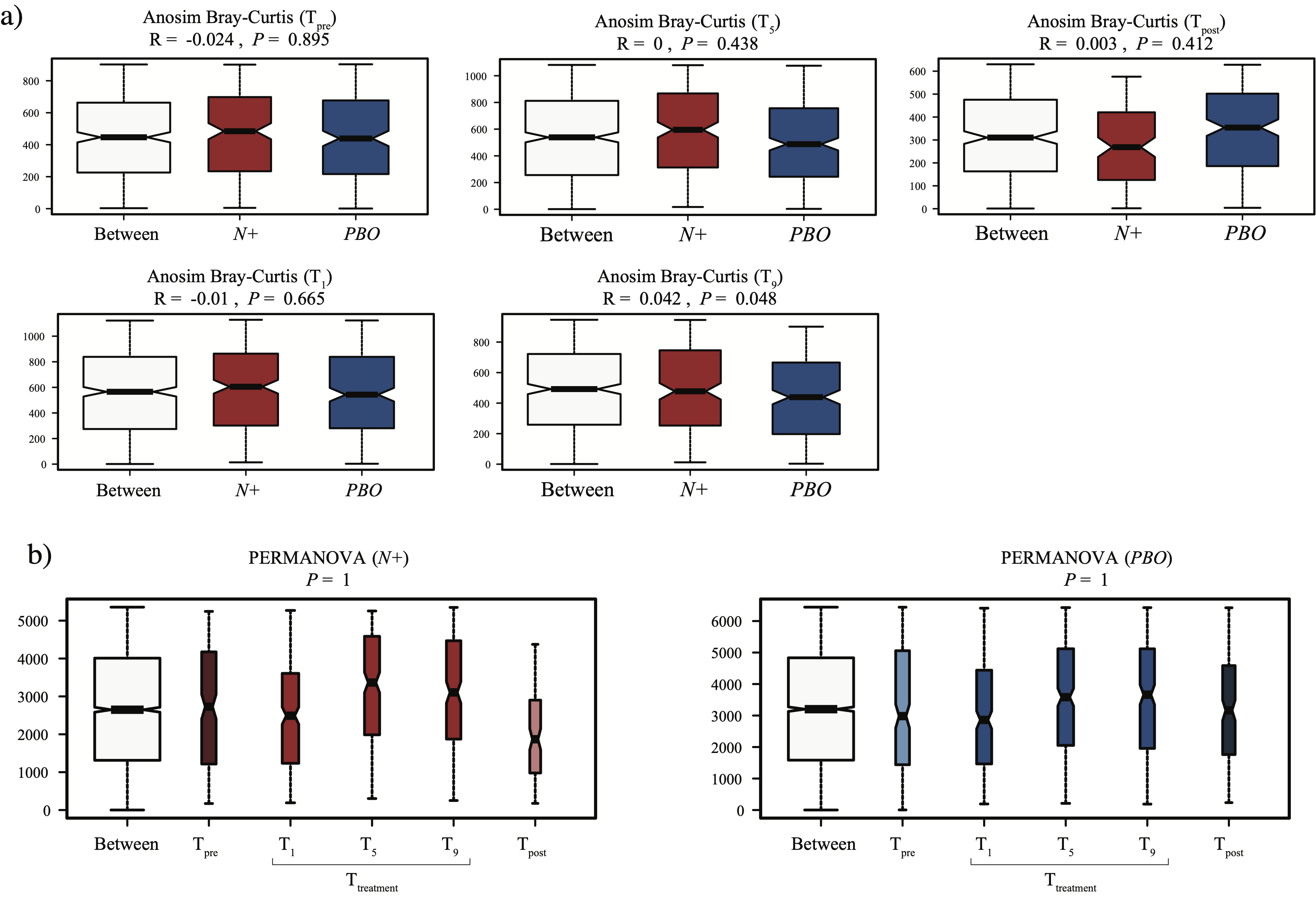

Supplement: Supplementary file 3 — Additional file 3. (a) Faecal bacterial beta diversity of volunteers with relapsing multiple sclerosis (RMS) experimentally infected with the hookworm, Necator americanus (N+), or placebo-treated (PBO) 1 week prior to infection/placebo treatment (Tpre), at 1, 5, and 9 months post-infection/placebo treatment (T1, T5 and T9, respectively), and 2 months post-anthelmintic treatment (Tpost; right). (b) Differences in faecal bacterial beta diversity between time points within N+ (left) and PBO (right) volunteers over the course of the study. ‘Between’ indicates the difference between groups (i.e. N+ and PBO [a] and Tpre, T1, T5, T9 and Tpost within each N+ and PBO [b]). [file 12915_2021_1003_MOESM3_ESM.png]

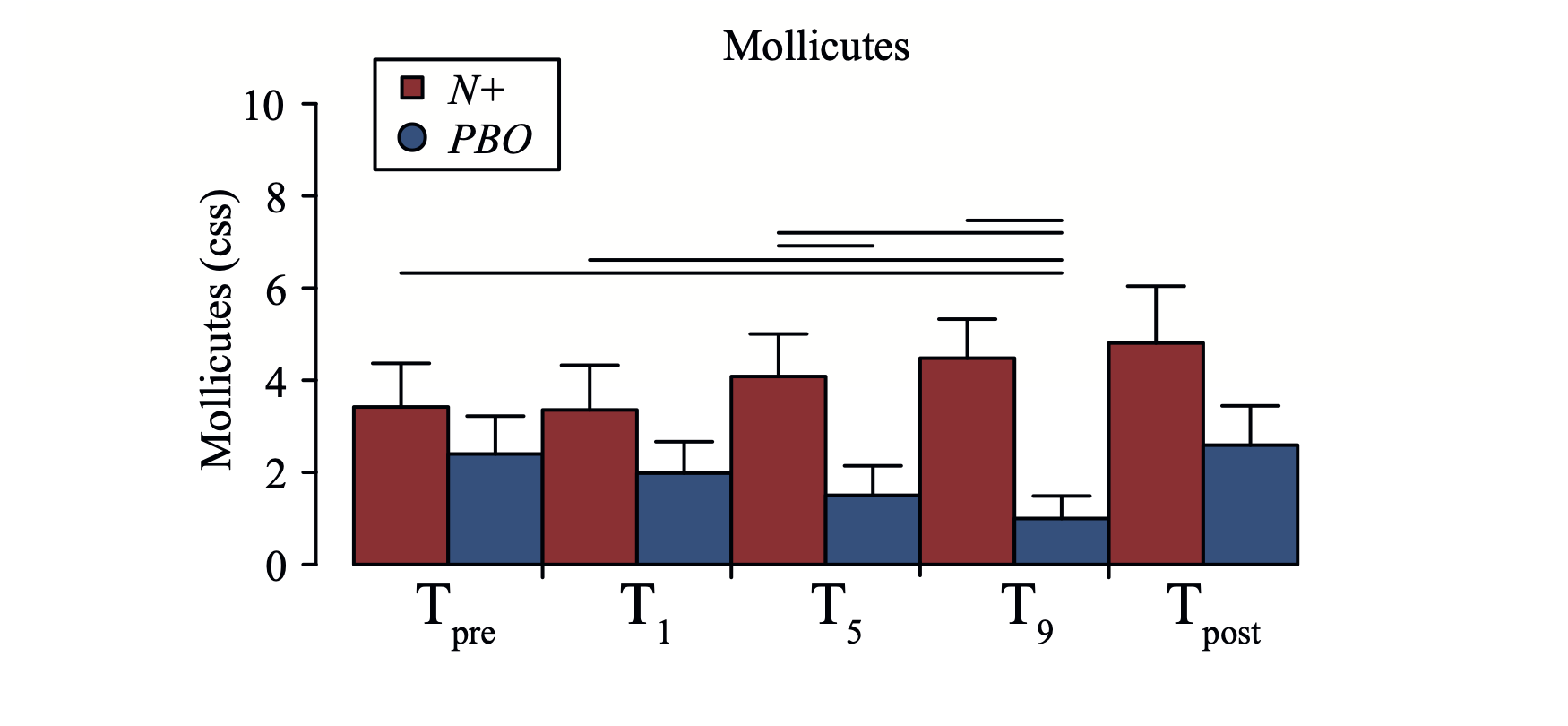

Supplement: Supplementary file 4 — Additional file 4. Differences in relative abundance of the bacterial class Mollicutes between the faecal microbiota of volunteers with relapsing multiple sclerosis (RMS) experimentally infected with the hookworm, Necator americanus (N+), or placebo-treated (PBO) across time points determined by ANOVA. Significant differences are indicated by horizontal bars (p < 0.05). [file 12915_2021_1003_MOESM4_ESM.png]

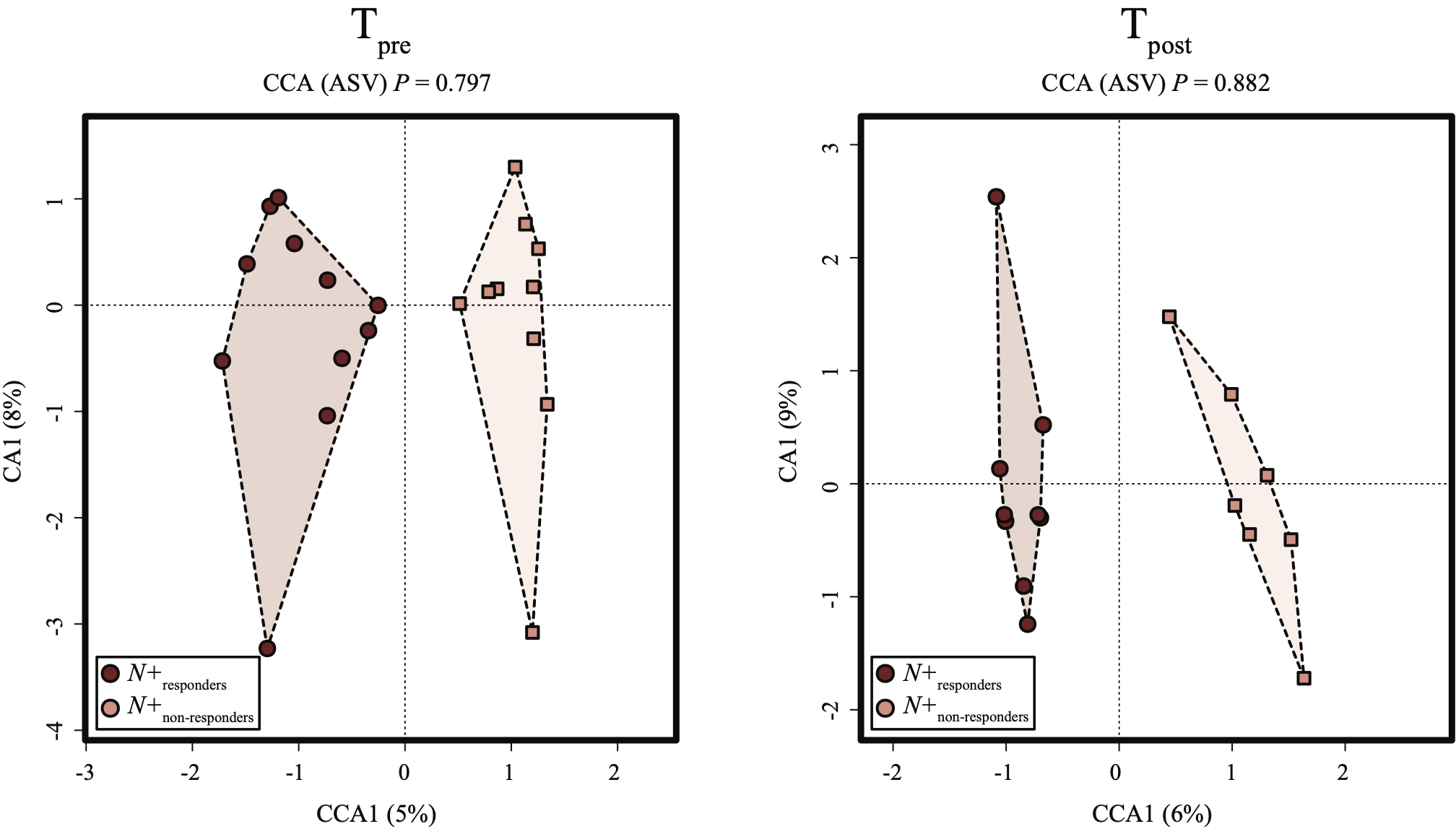

Supplement: Supplementary file 5 — Additional file 5. Differences in faecal bacterial profiles between Necator americanus-infected volunteers with relapsing-multiple sclerosis (RMS) who suffered a clinical and/or radiological relapse (N+non-responders) vs. volunteers for which no relapses were recorded (N+responders), investigated via supervised canonical correspondence analysis (CCA), prior to infection/placebo treatment (Tpre), as well as post-anthelmintic treatment (Tpost). [file 12915_2021_1003_MOESM5_ESM.png]

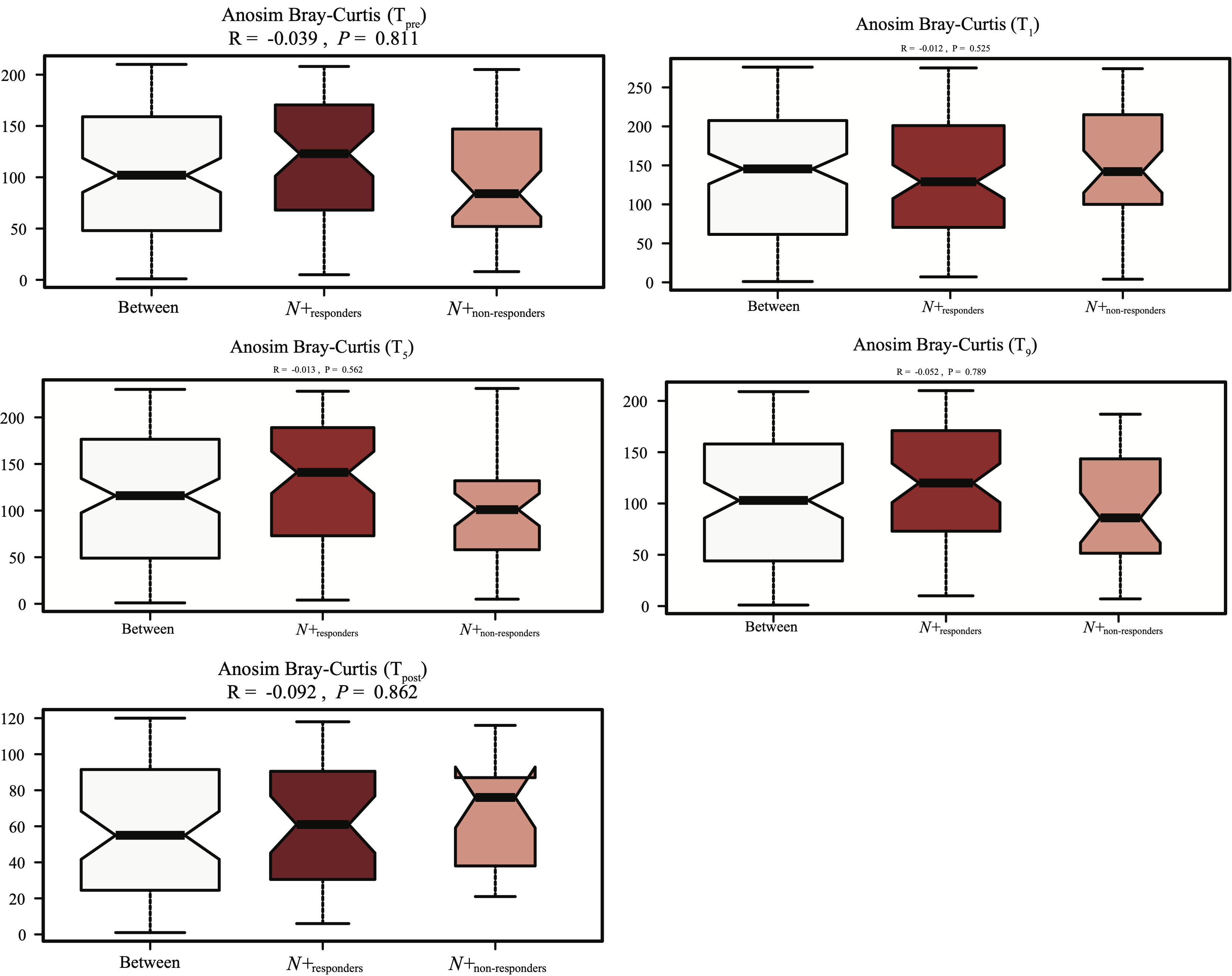

Supplement: Supplementary file 6 — Additional file 6. Analysis of similarities (ANOSIM) indicating differences in faecal bacterial beta diversity between Necator americanus-infected volunteers with relapsing multiple sclerosis (RMS) who suffered a clinical and/or radiological relapse (N+non-responders) vs. volunteers for which no relapses were recorded (N+responders), 1 week prior to infection/placebo treatment (Tpre), at 1, 5, and 9 months post-infection (T1, T5 and T9, respectively) and 2 months post-anthelmintic treatment (Tpost). Error bars are also provided. ‘Between’ indicates the difference between groups (i.e. N+responders and N+non-responders). [file 12915_2021_1003_MOESM6_ESM.png]

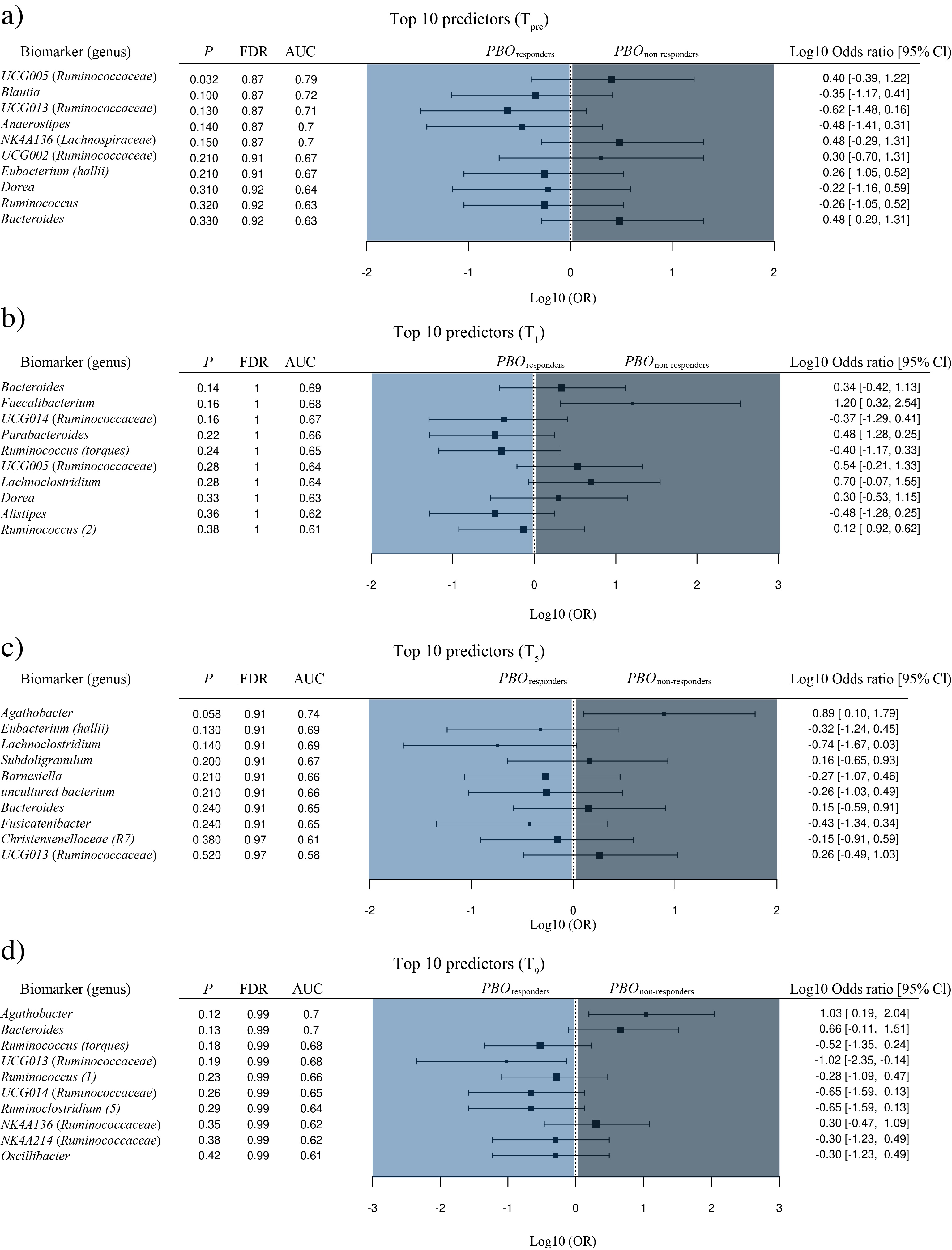

Supplement: Supplementary file 7 — Additional file 7. Top 10 faecal bacterial taxa identified as putative biomarkers of positive (PBOresponders) or negative (PBOnon-responders) clinical outcome for placebo-treated patients (PBO) over the course of the WIRMS trial. Biomarker predictions were conducted (a) one week prior to infection/placebo treatment (Tpre) and at (b) 1, (c) 5, and (d) 9 months post-infection (Ttreatment). [file 12915_2021_1003_MOESM7_ESM.jpg]
